# An odorant receptor mediates the avoidance of *Plutella xylostella* against parasitoid

**DOI:** 10.1186/s12915-024-01862-9

**Published:** 2024-03-13

**Authors:** Yipeng Liu, Sai Zhang, Song Cao, Emmanuelle Jacquin-Joly, Qiong Zhou, Yang Liu, Guirong Wang

**Affiliations:** 1 State Key Laboratory for Biology of Plant Diseases and Insect Pests, Institute of Plant Protection, Chinese Academy of Agricultural Sciences, Beijing, 100193 China; 2 Shenzhen Branch, Guangdong Laboratory for Lingnan Modern Agriculture, Genome Analysis Laboratory of the Ministry of Agriculture and Rural Affairs, Agricultural Genomics Institute at Shenzhen, Chinese Academy of Agricultural Sciences, Shenzhen, 518120 China; 3 Zhejiang Provincial Key Laboratory of Biometrology and Inspection and Quarantine, College of Life Sciences, China Jiliang University, Hangzhou, 310018 China; 4 Institute of Ecology and Environmental Sciences of Paris, INRAE, Sorbonne Université, CNRS, UPEC, UniversitéParis Cité, 78026 Versailles, IRD France; 5 College of Life Sciences, Hunan Normal University, Changsha, 410006 China

**Keywords:** *Plutella xylostella*, Odorant receptor, Heptanal, Avoidance behavior, Parasitoid wasp

## Abstract

**Background:**

Ecosystems are brimming with myriad compounds, including some at very low concentrations that are indispensable for insect survival and reproduction. Screening strategies for identifying active compounds are typically based on bioassay-guided approaches.

**Results:**

Here, we selected two candidate odorant receptors from a major pest of cruciferous plants—the diamondback moth *Plutella xylostella*—as targets to screen for active semiochemicals. One of these ORs, PxylOR16, exhibited a specific, sensitive response to heptanal, with both larvae and adult *P. xylostella* displaying heptanal avoidance behavior. Gene knockout studies based on CRISPR/Cas9 experimentally confirmed that PxylOR16 mediates this avoidance. Intriguingly, rather than being involved in *P. xylostella*–host plant interaction, we discovered that *P. xylostella* recognizes heptanal from the cuticular volatiles of the parasitoid wasp *Cotesia vestalis*, possibly to avoid parasitization.

**Conclusions:**

Our study thus showcases how the deorphanization of odorant receptors can drive discoveries about their complex functions in mediating insect survival. We also demonstrate that the use of odorant receptors as a screening platform could be efficient in identifying new behavioral regulators for application in pest management.

**Supplementary Information:**

The online version contains supplementary material available at 10.1186/s12915-024-01862-9.

## Background

In natural ecosystems, the most common communication channel between con-specific or hetero-specific insects and between insects and other living organisms is chemical communication, which depends on both volatile and non-volatile semiochemicals. Insects use such cues to guide a series of behaviors, such as courtship, mating, enemy avoidance, host positioning, and habitat selection [1, 2]. As early as 100 years ago, semiochemicals were used in pest control. Such semiochemicals are good choices for integrated pest management (IPM) strategies because of their strong selectivity, high efficiency, and environmental compatibility. Semiochemical-based pest control strategies reduce the use of chemical pesticides and the amount of pesticide residues in agricultural products, thus protecting health and preventing environmental harm [3–5]. Because of these advantages, semiochemicals have become a promising tool for agricultural pest management. At present, there are hundreds of semiochemicals used for population monitoring and pest control. With the continuous development of chemical ecology approaches, research into the interactions between insects and semiochemicals will yield better achievements, serving pest control and production practice.

The semiochemical identification process includes extraction or headspace collection, identification of active compounds, characterization of the chemical composition of the identified compounds, and elucidation of insect behavioral responses to the active semiochemicals. For decades, researchers have relied on conventional chemical ecology approaches based on electrophysiology such as electroantennography (EAG), gas chromatography–electroantennographic detection (GC-EAD), and indoor and/or field behavioral experiments to screen for active semiochemicals to be used in IPM [6, 7]. Such approaches have been successful in identifying sex pheromone lures for many moths and in developing strong bisexual kairomonal attractants for key crop pests such as the dried fruit beetle, the Mediterranean fruit fly, and the codling moth [8]. However, the development of new active semiochemicals requires extensive electrophysiological and behavioral biological assays, which are time-consuming, sometimes inefficient, and that always rely on the availability of living and healthy insects. Although GC-EAD enables convenient screening of active compounds, a large number of bioassay experiments are still required to avoid false positives [9]. For example, a compound can generate an electrical signal but be behaviorally inactive.

Knowledge of the molecular mechanisms underlying insect chemoreception opens innovative routes to identify active odorants via the so-called reverse chemical ecology approach, in which the molecular receptors of semiochemicals are targeted [9, 10]. These molecular receptors include multiple olfactory proteins, such as odorant-binding proteins (OBPs), chemosensory proteins, odorant receptors (ORs), ionotropic receptors, and sensory neuron membrane proteins [11–13]. OBPs and ORs have been the focus of previous studies because they are essential for volatile molecule signaling. For example, ORs regulate *Drosophila melanogaster* recognition of geosmin and pheromone and *Helicoverpa armigera* recognition of phenylacetaldehyde [14–16]. OBPs regulate *Ailuropoda melanoleuca* recognition of pheromones and *Culex quinquefasciatus* recognition of trimethylamine and nonanal [17, 18]. These examples show that ORs and OBPs can be used as targets to screen potential semiochemicals for other insects.

The diamondback moth, *Plutella xylostella* (Lepidoptera: Plutellidae), is a major pest of cruciferous plants and prefers to consume *Brassica* vegetables. Since 1990, the production of *Brassica* vegetable in China has increased 20-fold; meanwhile, the area of *Brassica* vegetable crops damaged by *P. xylostella* has also increased from 0.15 million ha in 1990 to 2.23 million ha in 2010; this pest is notorious for its strong reproductive ability and high insecticide resistance [19]. The annual crop losses and control costs due to *P. xylostella* represent 4–5 billion USD [20, 21]. In recent years, the use of semiochemicals has become the primary means for pest management, but there are still relatively few options for the control of *P. xylostella* because of a lack of candidate odorants [4, 19, 21–25]. Therefore, we performed an OR-based screen to identify new active odorants for possible application in the control *P. xylostella*, targeting two peculiar *P. xylostella* ORs (further referred as PxylORs) expressed in both larval and adult stages. We successfully identified an odorant, heptanal, whose detection is controlled by PxylOR16, and that mediates avoidance behavior of *P. xylostella* to the parasitoid wasp *Cotesia vestalis*. Our research not only paves the way for better understanding the mechanisms of the avoidance behavior to parasitoid wasps in this species but also highlights the efficient use of an OR to identify a behaviorally active volatile, providing an innovative approach for OR-based screening of potential behavioral regulators (repellents and attractants) for application in pest management.

## Results

### PxylOR16 is expressed in all the larval and adult stages of *P. xylostella* and specifically responds to heptanal

**Fig. 1:**
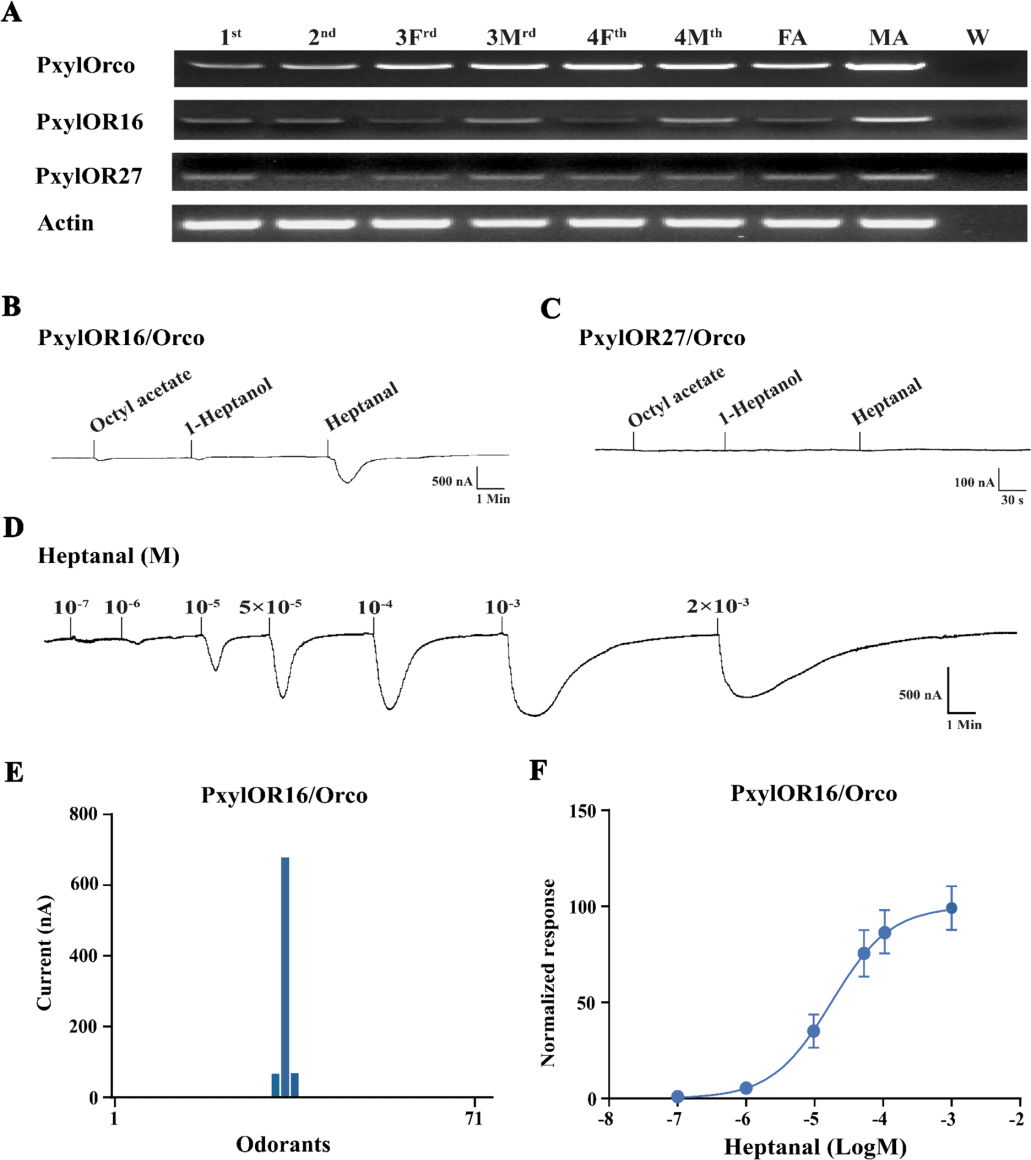
PxylOR16 is expressed in all the larval and adult stages of *Plutella xylostella* and is specifically tuned to heptanal. **A** Tissue expression patterns of *P. xylostella* OR genes. The cDNA templates for PCR analyses were from larval heads (first, second, third female, third male, fourth female, and fourth male instar larvae) and adult antennae (male adults: MA, female adults: FA). W water control. Among the 54 *P. xylostella* ORs, only *PxylOR16* and *PxylOR27* were detected in all larval stages and in adults. Actin was used for cDNA quality control. **B** Inward current responses of PxylOR16/PxylOrco *Xenopus* oocytes to plant volatile compounds (10^–4^ M). **C** No ligand was identified for PxylOR27/PxylOrco. **D** Inward current responses of PxylOR16/PxylOrco *Xenopus* oocytes stimulated with a range of heptanal concentrations. **E** Response profile of PxylOR16/PxylOrco *Xenopus* oocytes to a panel of 71 odorants (*n* = 6). **F** Dose–response curve of PxylOR16/PxylOrco *Xenopus* oocyte responses to heptanal. Heptanal EC_50_ = 1.757 × 10^–5^ M. Error bars indicate SEM (*n* = 6)

Most insect ORs are expressed in the adult antennae and, usually, only a few ORs are expressed in larvae. Some ORs expressed in both larvae and adults have been shown to exert key functions, for instance in *D. melanogaster*, *Spodoptera littoralis,* and *H. armigera* [16, 26, 27]. We thus searched for such ORs in *P. xylostella*, as targets for large screening to identify new active volatile compounds. First, the expression profile of 54 PxylORs in the heads (including antennae) of larvae at different instars (first, second, third female, third male, fourth female, and fourth male) and in the antennae of female and male adults were determined (Fig. 1A, Additional File 1: Fig. S1). The *P. xylostella* ortholog of the universal OR co-receptor (Orco), which is necessary for OR functioning, was found to be expressed in all samples examined. Most pheromone receptors (PRs) were specifically or highly expressed in the male antennae (*PxylOR1*, *PxylOR3*, *PxylOR4*, *PxylOR5*, *PxylOR6*, *PxylOR7*, and *PxylOR41*) (Additional File 1: Fig. S1).

Interestingly, only two ORs, *PxylOR16* and *PxylOR27*, were expressed in all examined larval stages and adult antennae of both sexes (Fig. 1A), suggesting they may have important biological functions throughout the insect life cycle. We subsequently expressed these two ORs in *Xenopus* oocytes to investigate their responsivity to a panel of 71 plant volatile compounds (Additional File 1: Table S1) using two-electrode voltage clamp. The oocytes expressing PxylOR16/Orco were extremely sensitive to heptanal, and they also responded weakly to two other volatiles (octyl acetate and 1-heptanol) (Fig. 1BE). In dose–response studies, we assayed the responses of PxylOR16 to a range of concentrations of heptanal and observed the lowest measurable response at a concentration of 1 × 10^−6^ M (Fig. 1D). The EC_50_ value of PxylOR16 for heptanal was 1.757 × 10^−5^ M (Fig. 1F). These results suggest that PxylOR16 and its ligand (heptanal) are of great significance to *P. xylostella*. Oocytes expressing PxylOR27 did not respond to any of the 71 examined plant volatile compounds (Fig. 1C).

### PxylOR16 knockout mutants show impaired electrophysiological responses to heptanal

**Fig. 2:**
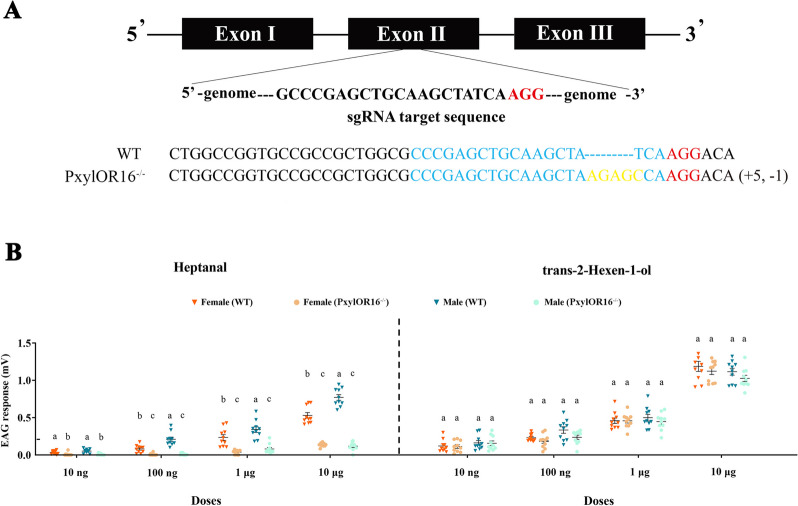
PxylOR16 knockout mutants show impaired electrophysiological responses to heptanal. **A** Schematic diagram of the sgRNA target in Exon II of *PxylOR16*. The target sequence is shown in blue, the PAM sequence is marked in red, and the non-homologous insertion in the genome in yellow. Deleted bases are represented by dashes. *PxylOR16* mutants show impaired electrophysiological responses to heptanal. **B** Electrophysiological responses measured as electroantennograms of *Plutella xylostella* antennae to heptanal and trans-2-hexen-1-ol in wild-type (WT) animals and in *PxylOR16* knockout mutants generated by CRISPR/Cas9 (*PxylOR16*^*−/−*^). Left, dose-dependent electroantennographic (EAG) responses of female and male moths. Heptanal was used at doses ranging from 10 ng to 10 μg. WT female and male antennae exhibited dose-dependent EAG responses to heptanal, with responses increasing with increasing heptanal doses. EAG responses of female and male *PxylOR16*^*−/−*^ moths to heptanal were far lower than those of WT female and male moths at all heptanal doses. Right, as a control, we tested the responses of female and male moths to trans-2-hexen-1-ol, which is not a ligand of PxylOR16. As expected, we observed no differences in the responses to this compound between *PxylOR16*^*−/−*^ moths and WT moths. Error bars indicate SEM (*n* = 10). Different letters indicate significant differences among insects (two-way ANOVA followed by Tukey’s pairwise test; *P* < 0.05)

Since heptanal appeared to strongly activate PxylOR16, we wondered whether this compound would also trigger a strong antennal response in vivo, as measured by EAG. Because the antennae of *P. xylostella* larvae are too small, we were not able to carry out EAG experiments on larvae. We thus recorded the electrophysiological responses of adult female and male antennae to heptanal doses of 10 ng, 100 ng, 1 μg, and 10 μg (Fig. 2B, left). Both female and male antennae displayed a dose-dependent EAG response to heptanal, with responses increasing with increasing heptanal doses. At the highest dose (10 μg), the EAG response values were maximal, with mean response values of 0.77 mV and 0.52 mV for wild-type females and males, respectively. At all doses except the lowest one (10 ng), the male EAG response values were significantly higher than those of females (Fig. 2B, left).We next constructed a PxylOR16-knockout strain using CRISPR/Cas9 genome editing in a traditional and common widely used method in many insects including *P. xylostella* [28], *H. armigera* [16]*, Locusta migratoria* [24, 29]*,* and *Eupeodes corollae* [30]. In all of these works, only one homozygous mutant strain was used. Therefore, knockout mutants (*PxylOR16*^*−/−*^) were obtained with a 5-nt insertion and a 1-nt deletion in the second exon, which introduced a premature stop codon in the coding sequence (Fig. 2A). This PxylOR16-knockout *P. xylostella* strain (*PxylOR16*^*−/−*^) was using for the further comparing of electrophysiological and behavioral responses with the wild-type (WT) strain. We also used EAG to investigate the electrophysiological response of *PxylOR16*^*−/−*^ moths to heptanal. As a control, we tested the response of female and male mutant moths to trans-2-hexen-1-ol, which is not a ligand of PxylOR16, and that is known evoke significant electrophysiological responses in the antennae of both female and male *P. xylostella* adults. As expected, we observed no difference between the responses of *PxylOR16*^*−/−*^ moths and WT moths to this compound (Fig. 2B, right). However, the EAG signals of female and male *PxylOR16*^*−/−*^ moths in response to heptanal were significantly reduced compared with those of WT female and male moths, at all examined heptanal doses (Fig. 2B, left). In the abovementioned results, the EAG responses to heptanal and trans-2-hexen-1-ol at different concentrations have been registered on the same individual, whether WT or *PxylOR16*^*−/−*^. These results support the hypothesis that PxylOR16 is involved in heptanal detection.

### Heptanal elicits obvious avoidance behaviors in *P. xylostella* larvae and adults

As *PxylOR16* was expressed in both larvae and adults, and having confirmed that PxylOR16 responded to heptanal, we next investigated the effect of this ligand on the behavior of *P. xylostella* adults and larvae. Behavioral experiments were conducted independently on female and male adults. Adult behavioral responses were tested using a Y-tube two-choice bioassay, while larvae responses were tested using a Petri dish assay (static air). As a control, we selected sex pheromone (Z-11-hexadecenal) to verify whether there is a difference in recognition of sex pheromones between the WT and *PxylOR16*^*−/−*^. The results of behavioral experiments showed that both WT and *PxylOR16*^*−/−*^ male moths were significantly attracted to Z-11-hexadecenal, and there was no significant difference between the two strains (Additional File 1: Fig. S2).

**Fig. 3:**
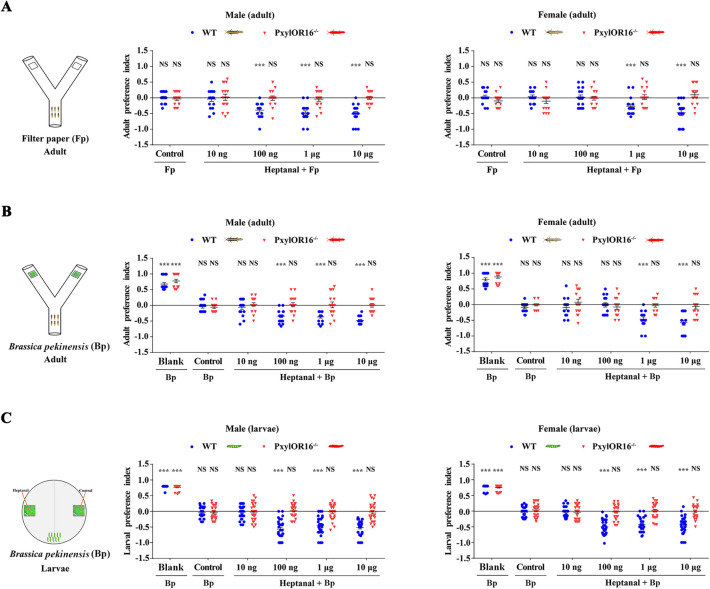
Heptanal elicits obvious avoidance behaviors in both *Plutella xylostella* larvae and adults. Preference index of *P. xylostella* wild-type (WT) and CRISPR/Cas9 PxylOR16 knockout (*PxylOR16*^*−/−*^) adults and larvae for heptanal. Preference indexes = (number of choices at treatment – number of choices at control) / number of total choices. **A** Preference index of WT and *PxylOR16*^*−/−*^ mutant female and male moths for control (filter paper vs filter paper) and for filter paper + paraffin oil vs filter paper + heptanal in a Y-tube olfactometer (*n* = 16). WT male moths exhibited significant avoidance to heptanal at doses of 100 ng, 1 μg, and 10 μg (left). Avoidance was also observed for WT female moths, but only at doses of 1 μg and 10 μg (Welch’s *t*-test; NS, no significant difference, *P* > 0.05; *** *P* < 0.001) (right). *PxylOR16*^*−/−*^ mutant female and male moths did not show a preference for one side of the device (Welch’s *t*-test; NS, no significant difference, *P* > 0.05). **B** Preference index of WT and *PxylOR16*^*−/−*^ mutant female and male moths for blank (one side blank vs a piece of *Brassica pekinensis*), for control (a piece of *B. pekinensis* vs a piece of *B. pekinensis*), and for a piece of *B. pekinensis* + paraffin oil vs a piece of *B. pekinensis* + heptanal in a Y-tube olfactometer (*n* = 16). WT and *PxylOR16*^*−/−*^ mutant female and male moths were significantly attracted to *B. pekinensis*, compared with blank. WT male moths were significantly less attracted to *B. pekinensis* + heptanal at doses of 100 ng, 1 μg, and 10 μg than to *B. pekinensis* alone (with paraffin oil) (left). Female moths were also less attracted to *B. pekinensis* + heptanal than to *B. pekinensis* alone but only at heptanal doses of 1 μg and 10 μg (Welch’s *t*-test; NS, no significant difference; *P* > 0.05; *** *P* < 0.001) (right). The *PxylOR16*^*−/−*^ mutant female and male moths lost their avoidance responses to heptanal significantly, compared with WT adult female and male moths (Welch’s *t*-test; NS, no significant difference; *P* > 0.05). **C** Preference indexes of 10 WT and mutant female and male third instar larvae for blank (one side blank vs a piece of *B. pekinensis*), for control (a piece of *B. pekinensis* vs a piece of *B. pekinensis*), and for a piece of *B. pekinensis* + paraffin oil vs a piece of *B. pekinensis* + heptanal in a 10-cm diameter plastic Petri dish (*n* = 28). Both sexes of WT and mutant larvae were attracted to *B. pekinensis*. Significantly more female and male third instar larvae were located in the control area (*B. pekinensis* + paraffin oil) than in the heptanal-supplemented area (*B. pekinensis* + heptanal) at all heptanal doses (100 ng, 1 μg, and 10 μg on filter paper) except for 10 ng (Welch’s *t*-test; NS, no significant difference, *P* > 0.05; *** *P* < 0.001). As shown for *PxylOR16*^*−/−*^ adults, detection of heptanal was abolished in *PxylOR16*^*−/−*^ larvae (Welch’s *t*-test; NS, no significant difference, *P* > 0.05)

According to the Y-tube two-choice bioassay for adults, we first validated this assay by performing control experiment in which insects were exposed to filter paper without any odorant added on both sides. We observed that neither WT nor *PxylOR16*^*−/−*^ adults displayed any selection preference (Fig. 3A). Then, we tested adult behavioral responses to heptanal at different concentrations. The experiment revealed that male moths displayed significant avoidance to heptanal compared with the paraffin oil control at doses of 100 ng, 1 μg, and 10 μg (Fig. 3A, left). The same avoidance behavior was observed for female moths, but only at doses of 1 μg and 10 μg (Fig. 3A, right), suggesting that female and male moths have different sensitivities to heptanal, in accordance with the EAG results.

Then, we performed a second experiment on adults using the Y-tube two-choice bioassay to test the response of *P. xylostella* adults to heptanal in the presence of the host *Brassica pekinensis* leaf (1 × 1 cm^2^, the oviposition material for adults used in our laboratory) as the attractant in both tube arms. In the one side blank experiment, we added a piece of *B. pekinensis* in one arm and kept the other arm blank. Both WT and *PxylOR16*^*−/−*^ adults exhibited a preference for *B. pekinensis* over the blank (Fig. 3B, Blank). This result showed that the *B. pekinensis* leaf was attractive to adults, whatever the genotype. When adults were challenged with *B. pekinensis* on both sides, neither WT nor *PxylOR16*^*−/−*^ adults displayed any selection preference (Fig. 3B, Control). When we compared the choice between *B. pekinensis* and *B. pekinensis* + heptanal, we found that WT male moths were significantly less attracted to *B. pekinensis* + heptanal than to *B. pekinensis* alone (with paraffin oil), at heptanal doses of 100 ng, 1 μg, and 10 μg (Fig. 3B, left). Female WT moths were also less attracted to *B. pekinensis* + heptanal than to *B. pekinensis* alone, but only at heptanal doses of 1 μg and 10 μg (Fig. 3B, right), indicating that male moths were still more sensitive to heptanal than female moths in the presence of *B. pekinensis*. Consistent with our finding that PxylOR16 mediates *P. xylostella* perception of heptanal, *PxylOR16*^*−/−*^ adults in the Y-tube two-choice bioassay showed no preference under any examined heptanal concentration (Fig. 3AB).

Larvae behavioral responses were tested using a Petri dish assay (static air). As for the Y-tube assay conducted in adults, we first conducted a control one side blank experiment in which a piece of *B. pekinensis* (1 × 1 cm^2^, the food source for larvae used in our laboratory) was added on one side of the dish, while keeping the other side blank. This result showed the food source was also attractive to larvae (Fig. 3C, Blank). When larvae were challenged with *B. pekinensis* on both sides, neither WT nor *PxylOR16*^*−/−*^ larvae displayed any selection preference (Fig. 3C, Control). In view of these results, we used the Petri dish assay to test the response of *P. xylostella* larvae to heptanal (heptanal alone vs. paraffin oil control) in the absence of food (*B. pekinensis*) and found that *P. xylostella* larvae did not make effective choices. We next tested the response of *P. xylostella* larvae to heptanal in the presence of a piece of *B. pekinensis* using the Petri dish assay. Significantly more female and male third instar larvae were located in the control area (*B. pekinensis* + paraffin oil) than in the heptanal-supplemented area (*B. pekinensis* + heptanal) at all heptanal doses (100 ng, 1 μg, and 10 μg on filter paper) except at 10 ng (Fig. 3C). To verify whether PxylOR16 was responsible for this effect in the larvae, we challenged *PxylOR16*^*−/−*^ larvae in the Petri dish assay. As shown for *PxylOR16*^*−/−*^ adults, detection of heptanal was abolished in *PxylOR16*^*−/−*^ larvae (Fig. 3C). Taken together, these results suggest that PxylOR16 regulates the heptanal avoidance behavior of *P. xylostella* larvae and adults.

### Heptanal does not participate in the direct interaction between *P. xylostella* and its host plant

Heptanal is a common plant volatile, so we firstly speculated that heptanal may mediate interactions between *P. xylostella* and host plants. We conducted a series of behavioral experiments to test if heptanal participates in the direct interaction between *P. xylostella* and *Brassica* vegetables. We chose* Brassica* plants for experiments because it is the most important genus of cruciferous plants and also the favorite host plant of *P. xylostella*. In the past 20 years, *P. xylostella* has become the most destructive insect pest on *Brassica* vegetables [21]. Therefore, we selected a common *Brassica* vegetable (*B. parachinensis*) for the following experiments.

**Fig. 4:**
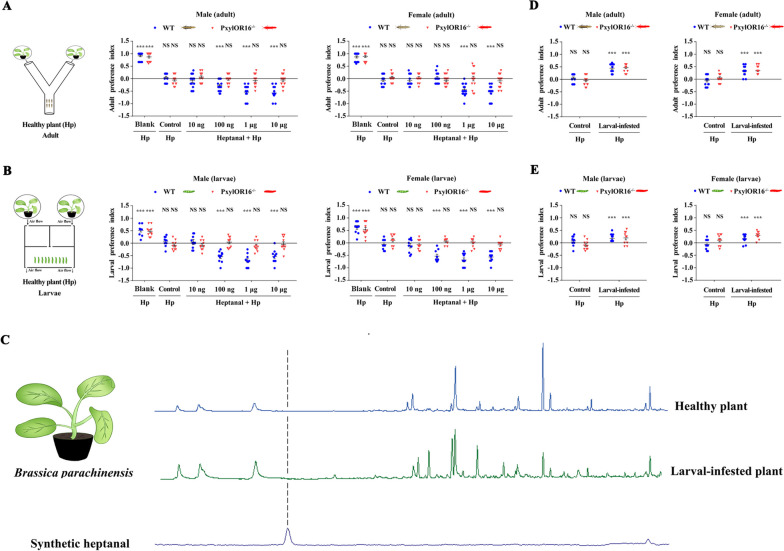
Heptanal does not participate in the direct interaction between *Plutella xylostella* and its host plant. Preference index of *P. xylostella* wild-type (WT) and PxylOR16-knockout (*PxylOR16*^*−/−*^) adults and larvae to heptanal. **A** Preference index of WT and *PxylOR16*^*−/−*^ mutant female and male moths for blank (one side blank vs an intact healthy *Brassica parachinensis* plant), for control (an intact healthy *B. parachinensis* plant vs an intact healthy *B. parachinensis* plant), and for an intact healthy *B. parachinensis* plant + paraffin oil vs an intact healthy *B. parachinensis* plant + heptanal in a Y-tube olfactometer (*n* = 16). WT and *PxylOR16*^*−/−*^ mutant female and male moths were significantly attracted to healthy *B. parachinensis* plant, compared with blank. Three doses (100 ng, 1 μg, and 10 μg) and two doses (1 μg and 10 μg) of heptanal elicited avoidance behavior in WT female and male *P. xylostella* adults, (Welch’s *t*-test; NS, no significant difference, *P* > 0.05; *** *P* < 0.001). The numbers of *PxylOR16*^−/−^ adults on each side of the Y-tube olfactometer were not significantly different at all heptanal doses tested, when comparing *B. parachinensis* + heptanal with *B. parachinensis* plant + paraffin oil (Welch’s *t*-test; NS, no significant difference, *P* > 0.05). **B** Preference indexes of 10 WT and mutant female and male third instar larvae for blank (one side blank vs an intact healthy *B. parachinensis* plant), for control (an intact healthy *B. parachinensis* plant vs an intact healthy *B. parachinensis* plant), and for an intact healthy *B. parachinensis* plant + paraffin oil vs an intact healthy *B. parachinensis* plant + heptanal in a screening device (*n* = 10). Both sexes of WT and mutant larvae were attracted to the *B. parachinensis* plant. WT female and male larvae (third instar larvae) preferred the healthy plant with paraffin oil to a healthy plant with heptanal at three doses (100 ng, 1 μg, and 10 μg), unlike *PxylOR16*^−/−^ larvae (Welch’s *t*-test; NS, no significant difference, *P* > 0.05; *** *P* < 0.001). **C** GC–MS analysis of the volatiles produced by larval-infested plants and healthy plants; heptanal was not detected. Larval-infested *B. parachinensis* (top), healthy *B. parachinensis* (middle), and synthetic heptanal (bottom). **D** Preference index of WT and *PxylOR16*^*−/−*^ mutant adults for control (intact healthy *B. parachinensis* plant vs intact healthy *B. parachinensis* plant) and for an intact healthy *B. parachinensis* plant vs a larval-infected *B. parachinensis* plant in a Y-tube olfactometer (*n* = 16). There was no difference in selection between the WT and *PxylOR16*^*−/−*^ mutant; both preferred the larval-infected plant (Welch’s *t*-test; NS, no significant difference, *P* > 0.05; *** *P* < 0.001). **E** Preference index of WT and *PxylOR16*^*−/−*^ mutant larvae for control (intact healthy *B. parachinensis* plant vs intact healthy *B. parachinensis* plant) and for an intact healthy *B. parachinensis* plant vs a larval-infected *B. parachinensis* plant in a screening device (*n* = 10). Like adults, WT and *PxylOR16*^*−/−*^ mutant larvae both preferred the larval-infected plant (Welch’s *t*-test; NS, no significant difference, *P* > 0.05; *** *P* < 0.001)

The effect of heptanal in the presence of the host plant *B. parachinensis* on the adults and larvae were tested by using Y-tube two-choice bioassay and a screening device (with airflow) separately. The responses of *P. xylostella* adults and larvae to a healthy *B. parachinensis* plant were tested in one side blank experiments. The healthy *B. parachinensis* plant did have a significant attracting effect on both WT and *PxylOR16*^*−/−*^ adults and larvae, and neither WT nor *PxylOR16*^*−/−*^ insects displayed any preference in control experiments when *B. parachinensis* was present in both sides (Fig. 4AB).

In subsequent experiments, female and male moths were given a choice between an intact *B. parachinensis* plant without and with different doses of heptanal in a Y-tube assay. Three doses (100 ng, 1 μg, and 10 μg) and two doses (1 μg and 10 μg) of heptanal elicited avoidance behaviors in female and male *P. xylostella* adults, respectively (Fig. 4A). In larvae, we observed a preference of female and male larvae (third instar larvae) for a healthy *B. parachinensis* plant with paraffin oil over a healthy plant with heptanal at three doses (100 ng, 1 μg, and 10 μg) (Fig. 4B). The numbers of *PxylOR16*^−/−^ adults and larvae on each side of the device were not significantly different at all heptanal doses tested (Fig. 4AB). To test whether heptanal is present in the host plant *B. parachinensis*, we examined the volatiles emitted by the healthy *B. parachinensis*. Briefly, we used solid-phase microextraction to collect volatiles emitted by healthy plants. Gas chromatography coupled to mass spectrometry (GC/MS) analysis revealed that no heptanal was detectable in healthy *B. parachinensis* volatiles (Fig. 4C).

We found that an intact *B. parachinensis* plant had a significant attracting effect on *P. xylostella* and that its presence did not affect the heptanal avoidance behavior, so we further tested if the *B. parachinensis* plants damaged by *P. xylostella* feeding will release heptanal and evoke avoidance behavior. Female and male moths and third instar larvae were given a choice between an intact *B. parachinensis* plant and larval-infected *B. parachinensis* plant (larvae and feces were removed, same treatment in following experiments) in a Y-tube and in a screening device: the adults and larvae had a preference for larval-infected plants compared with heathy plants (Fig. 4DE). These results were not unexpected, since *P. xylostella* was previously reported to prefer *P. xylostella* larval-infected plants [31, 32]. Moreover, like the WT, the *PxylOR16*^−/−^ adults and larvae also showed a preference for larval-infected plants (Fig. 4DE). Next, we also examined the volatiles emitted by the *P. xylostella* larval-infested *B. parachinensis*. GC/MS analysis revealed that no heptanal was detectable in larval-infested *B. parachinensis* volatiles (Fig. 4C).

These results suggest that heptanal cannot explain the direct interaction between *P. xylostella* and *B. parachinensis* (intact or pest infested). Similarly, many other *Brassica* plants do not release heptanal [33], so we speculate that heptanal is not involved in the interactions between *P. xylostella* and host plants as a plant volatile.

### *P. xylostella* larvae and adults detect and avoid *Cotesia vestalis* odorants

Heptanal was previously reported to be a cuticular volatile emitted and sensed by some *Cotesia* parasitic wasp species, including *C. glomerata* and *C. marginiventris*, and this compound is a required component of their sex communication system [34, 35]. Both females and males of *C. glomerata*, a parasitic wasp of *Pieris brassicae*, release heptanal as a repellent pheromone. After mating, females release heptanal to repel males to avoid mating again, while the males release heptanal to repel conspecific males, thus reducing male–male competition on the natal patch and ensuring maximal mating success. The amount of heptanal released by *C. glomerata* was analyzed. It was previously found that about 3 ng can be obtained from 6 individuals by solvent extraction, and that about 50 ng can be obtained from 200 individuals by headspace extraction [34]. In a phylogeny of 25 species of *Cotesia* commonly used in laboratory and field research, *C. vestalis* is the closest to *C. glomerata* [36]. We therefore turned our attention to *C. vestalis*, which is an endoparasitoid wasp that is known to attack *P. xylostella* larvae; indeed, it is used as an agricultural control measure against *P. xylostella* [37].

**Fig. 5:**
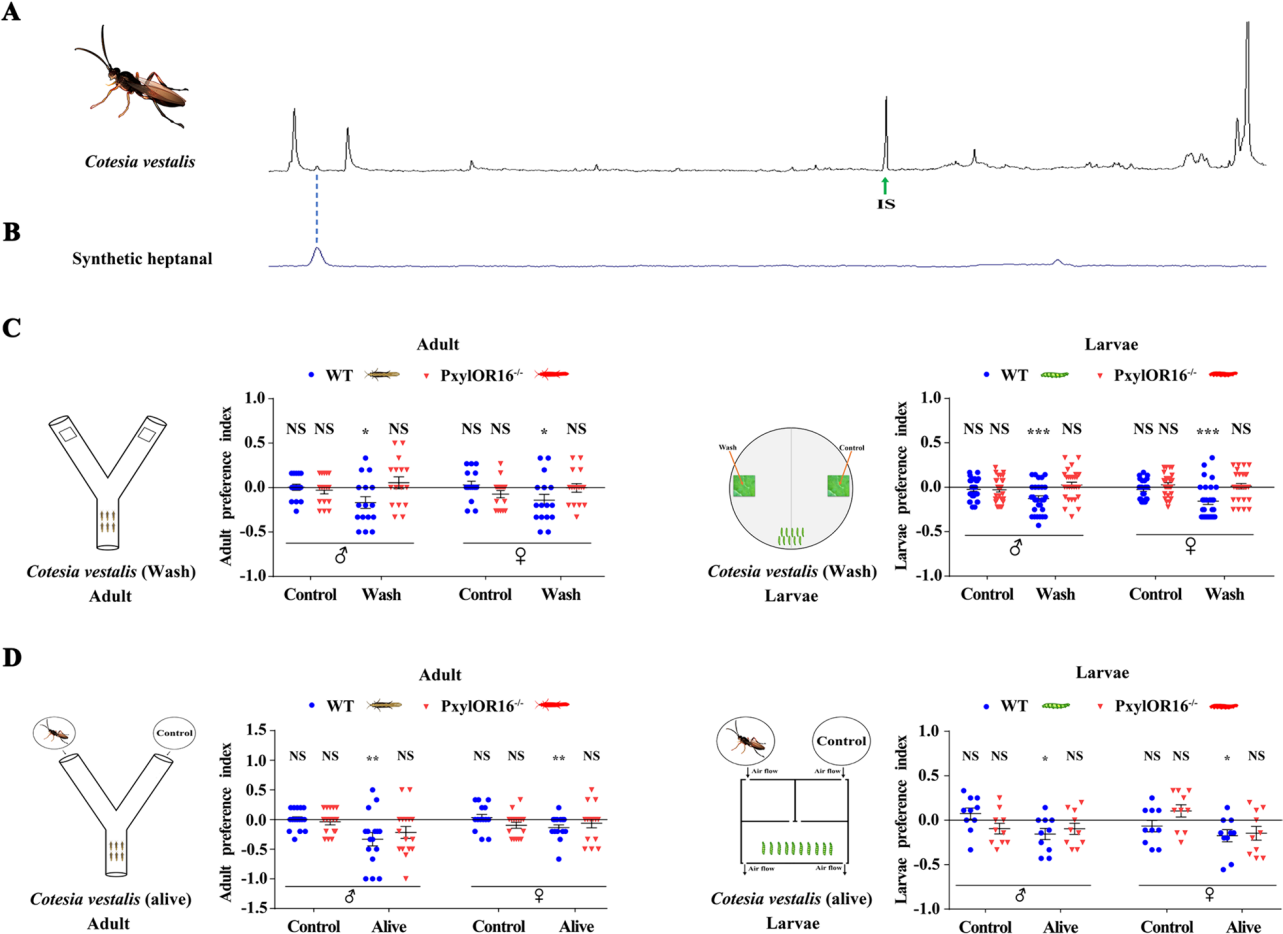
*Plutella xylostella* larvae and adult detect and avoid *Cotesia vestalis* odorants. **A** GC/MS analysis of the volatiles produced by *C. vestalis*. Heptanal could be detected in extracts of *C. vestalis*. IS internal standard. **B** Synthetic heptanal. **C** Preference index of WT and *PxylOR16*^*−/−*^* P. xylostella* mutant adults (left) and larvae (right) for filter paper + dichloromethane vs filter paper + *C. vestalis* body wash in a Y-tube olfactometer and a 10-cm diameter plastic Petri dish, respectively (*n* = 16, adults;* n* = 28, larvae). Both sexes of WT larvae and adults of *P. xylostella* had avoidance responses to a *C. vestalis* body wash, but the *PxylOR16*^*−/−*^ mutant did not (Welch’s *t*-test; NS, no significant difference; *P* > 0.05; * *P* < 0.05; *** *P* < 0.001). **D** Preference index of WT and *PxylOR16*^*−/−*^ mutant adults (left) and larvae (right) for air + 20 live *C. vestalis* vs air in a Y-tube olfactometer and a screening device, respectively (*n* = 16, adults; *n* = 10, larvae). Both sexes of WT larvae and adults of *P. xylostella* showed avoidance of *C. vestalis*, but the *PxylOR16*^*−/−*^ mutants did not (Welch’s *t*-test; NS, no significant difference; *P* > 0.05; * *P* < 0.05; ** *P* < 0.01)

We hypothesized that, like *C. glomerata*, *C. vestalis* releases heptanal. To test this hypothesis, we collected cuticular compounds of this wasp using dichloromethane for GC/MS analysis. Consistent with the hypothesis that *C. vestalis* releases heptanal, we could detect heptanal in the extracts of a mixture of 10 female and 10 male wasps, and the amount collected from these twenty wasps was 2.47 ± 0.55 ng (Fig. 5AB).

Both *D. melanogaster* adults and larvae have an avoidance response to *Leptopilina boulardi*, which is a parasitic wasp of *D. melanogaster* larvae [26]. To test whether larvae and adults of *P. xylostella* have a similar avoidance response to *C. vestalis* odorants, we next conducted a behavioral experiment in two different experimental treatments: (1) a *C. vestalis* body wash obtained by extracting 20 wasps (females and males) with dichloromethane for 30 min and (2) 20 live *C. vestalis* wasps (females and males) placed in glass jars connected by a Y-tube or a screening device. For the first experimental treatment, a Y-tube two-choice bioassay and a Petri dish assay revealed that a *C. vestalis* body wash elicited avoidance responses in adults (Fig. 5C, left) and in larvae (Fig. 5C, right). Both adults (Fig. 5D, left) and larvae (Fig. 5D, right) of *P. xylostella* also showed avoidance behavior in response to live *C. vestalis* in a Y-tube two-choice bioassay and using a screening device.

In behavioral experiments using *PxylOR16*^*−/−*^, we found that both *PxylOR16*^*−/−*^ larvae and adults no longer had an avoidance response to *C. vestalis* body wash or live *C. vestalis* (Fig. 5CD). These results show that *P. xylostella* larvae and adults use PxylOR16 to detect heptanal released by *C. vestalis* and thereby to regulate their avoidance to *C. vestalis*.

## Discussion

In this study, we identified two *P. xylostella* OR expressed at all developmental stages. One of these ORs, PxylOR27, did not respond to any of the 71 examined plant volatile compounds. We thus speculate that the ligand(s) of PxylOR27 do not belong to the compound panel we tested here or that PxylOR27 did not successfully express in oocyte membrane [16, 38]. The other OR, PxylOR16 expressed at all developmental stages and detected under the sensilla trichoidea of male and female antennae (Additional File 1: Fig. S3), was activated by heptanal. This receptor appeared to be narrowly tuned, a characteristic of ORs tuned to ecologically relevant stimuli [15, 38, 39] and involved in labeled line olfactory circuits [40]. We thus suspected that heptanal may play an important role in *P. xylostella* and searched for its function.

We investigated whether heptanal could be an indicator of an unsuitable environment for *P. xylostella*. Heptanal is known to have different functions in multiple species. It has been identified as a component of linden (*Tilia tuan*), which can elicit electroantennographic and behavioral responses in *Agrotis ipsilon*. Heptanal is an important compound released by peach flowers and mature fruits, and it also elicits electroantennographic responses in *Grapholita molesta* and attracts this species [41–43]. Similarly, heptanal is a novel identified pheromone, which is derived from the cuticular hydrocarbons of *D. melanogaster* and plays an important role in the evaluation of oviposition site [44]. Heptanal can also have different bioactivities depending on its concentration. For example, in *Phthorimaea operculella*, it acts as an oviposition repellent at high concentrations and as an attractant at low concentrations [45]. Interestingly, most cruciferous plants—the host plants of *P. xylostella*—do not release heptanal [33]. We found that neither healthy nor larval-infested *Brassica* could repel *P. xylostella.* On the contrary, *P. xylostella* larvae and adults preferred larval-infested plants over healthy plants, which is consistent with previous studies [31, 32]. This is because the release of isothiocyanates increases after *Brassica* are damaged, and isothiocyanates are highly attractive to *P. xylostella* [46, 47]. Thus, the ecological significance of the avoidance behavior elicited by heptanal does not lie in the interaction between *P. xylostella* and *Brassica*, but may be related to the detection of other dangers, such as the presence of natural enemies.

We then searched for other sources of heptanal in the *P. xylostella* environment. We focused on natural enemies such as endoparasitoid wasps, since heptanal is known to be emitted by some *Cotesia* species. Heptanal has been shown to function directly in the pheromonal communication of *C. glomerata* and *C. marginiventris* [34, 35]. Our experiments showed that larvae and adults of *P. xylostella* avoid heptanal. This shows that among *P. brassicae*–*C. glomerata*–*P. xylostella* in the same ecosystem, heptanal belongs to a “semiochemical parsimony”, which refers to the use of a chemical substance for two or more purposes [48]. Another reason for the release of heptanal by *C. glomerata* may be to protect *P. brassicae*, leading to a better feeding environment. A similar situation also occurs in the interaction among *D. melanogaster*, *Leptopilina heterotoma*, and ants. *L. heterotoma* is a parasitic wasp of *D. melanogaster* and releases iridomyrmecins to repel ants; this defense mechanism allows the parasitic wasp to forage without interference from the ants, thereby improving their parasitism and reproductive success. At the same time, iridomyrmecins also take part in the mating behavior of *L. heterotoma* [49, 50].

For instance, *C. marginiventris* females can release heptanal to significantly improve their attractiveness to males [35]. *C. vestalis* is a *Cotesia* species known to be an endoparasitoid of *P. xylostella*. Heptanal is an attractant for *C. vestalis*, and this attractant has been used successfully in the biological control of *P. xylostella* [37]. We thus hypothesized that *C. vestalis* may also release heptanal, and we confirmed this hypothesis by revealing that this compound was a component of a *C. vestalis* cuticular extract. Taken together our results, we propose that heptanal serves as a signal for both *P. xylostella* adults and larvae to recognize natural enemies such as *C. vestalis* and avoid parasitization. Further experiments, such as testing the effect of heptanal and the role of PxylOR16 on the parasitism rate of *P. xylostella* in the presence of *C. vestalis*, would definitely confirm this hypothesis. It has to be noticed that *P. xylostella* avoidance behavior was observed at high wasp density (20 wasps were needed to elicit avoidance). One explanation could be that a trade-off exists between the need to feed/oviposit and parasitization risk: at low wasp concentration, feeding or oviposition would be prioritized, whereas at high was concentration, escape is prioritized.

Nevertheless, our results suggest an essential purpose of heptanal in *Cotesia* parasitoids as it is released by at least three different *Cotesia* species. There are more than 350,000 species of parasitoid wasps on earth [51], indicating that they are an indispensable part of the self-regulation of the ecosystem. Insects have evolved a series of defenses, some cellular and some behavioral, to protect themselves against these parasitoids [52, 53]. *D. melanogaster* regulates defensive behaviors against parasitic wasps through different kinds of neurons. For example, nociceptive neurons mediate rolling behavior [49], visual cues regulate oviposition behavior [54, 55], and olfactory neurons detect odors of parasitic wasps and drive aversion responses [26]. Studies have shown that the presence of certain parasitoid wasps can affect the mating behavior of *D. melanogaster* [56]. In addition, the insect olfactory system plays an important role in the detection of parasitic wasps, and there are many examples of species using allomones produced by natural enemies to escape or to choose habitats without, or that are not favorable to, natural enemies to feed and lay eggs [26, 57, 58]. Therefore, accurately determining the presence or absence of natural enemies is crucial to the life activities of insects. *P. xylostella* may use heptanal as a primary indicator for judging the presence of *C. vestalis* or *C. glomerata* in the same ecological environment. The use by a species of odors released by another species for sexual communication to avoid natural enemies is highly adaptive because the odor-based sex communication system cannot be easily changed [26]. The phenomenon of insects using odorants of enemies for avoidance is in line with the strategy used by *P. xylostella* against its enemy.

Our study provides a reference for OR-based screening of novel behavioral regulators of *P. xylostella*, which could serve as a scientific basis for formulating and improving integrated pest control strategies. In addition, we gained a deeper understanding of how insect pests recognize volatiles produced by their natural enemies through olfaction. Better knowledge of the relationship between the host plants, parasitoid wasps, heptanal, and *P. xylostella* is now needed to understand the balance between preference and repellency, especially in females. Such knowledge would be useful in the control of *P. xylostella* infestation by enhancing indirect plant defense via modification of the heptanal synthetic pathway or by using adequate amounts of synthetic heptanal to repel *P. xylostella*.

## Conclusions

We identified heptanal as a repellent for both larvae and adults of *P. xylostella*, via the functional characterization of the odorant receptor PxylOR16. Our findings shed new insights on the interaction mechanism among insect pests and their natural enemies and provides a candidate compound that could be used for integrated pest management of this important agricultural pest.

## Methods

### Plants and insect rearing

*P. xylostella* larvae were obtained from a colony maintained at the Institute of Plant Protection, Chinese Academy of Agricultural Sciences, which was collected from a suburban area in Beijing in 2001. The larvae were reared on *B. pekinensis*, and the adults were fed with 10% honey water at 26 ± 2 ℃ on a 16:8-h (light/dark) photoperiod cycle and 50–70% relative humidity (RH). *C. vestalis* cocoons were obtained from the Institute of Insect Sciences, Zhejiang University, China. Adult wasps were propagated using third instar *P. xylostella* larvae, and the rearing of *C. vestalis* colonies was conducted following previously reported protocols [59]. Seedlings of *B. parachinensis* Bailey were cultivated in a growth chamber (25 ± 2 ℃, 50–70% RH, 12:12 light/dark regime) for 5–6 weeks up to the six-leaf stage.

### Expression analyses

Larval heads (first, second, third female, third male, fourth female, and fourth male instars) and female and male adult antennae of *P. xylostella* were collected. It is easy to distinguish the sexes of third and fourth instar larvae, as male larvae have testes in the fifth abdominal segment, and their backs are pale yellow. Total RNAs were extracted separately from each collected tissue using TRIzol Reagent (Invitrogen, Carlsbad, CA) and treated with DNaseI to remove trace amounts of genomic DNA. First-strand cDNAs were synthesized using the RevertAid First Strand cDNA Synthesis Kit (Fermentas, Glen Burnie, MD, USA) and were used as templates RT-PCR. The expression patterns of all PxylORs were investigated. The primer sequences are listed in Additional File 1: Table S2. PCR conditions: 94 ℃ for 3 min, then 35 (larvae) or 26 (adults) cycles at 94 ℃ for 30 s, 55 ℃ for 40 s, and 72℃ for 60 s, with a final 10-min incubation at 72 ℃. PCR products were analyzed on 2.0% agarose gels. The experiments were repeated three times with independent RNA samples.

### Cloning of PxylOR16 and PxylOR27

The open reading frames of *PxylOR16* and *PxylOR27* were cloned using specific primers (Additional File 1: Table S2). PCR amplification products were purified from 1% agarose gels and ligated into the pEASY-Blunt vector (TransGenBiotech, China). After transformation of Trans1-T1 competent cells (TransGenBiotech, China), selected positive clones were sequenced by BGI (Beijing, China).

### Plant volatile compounds

Seventy-one odorants, including host–plant volatiles and known behaviorally and physiologically active semiochemicals, were used for functional characterization of PxylORs. All compounds were purchased from Sigma-Aldrich (purity ≥ 98%) and are listed in Additional File 1: Table S1. For functional analyses of PxylORs using two electrode voltage clamp electrophysiological recording, compounds were diluted to 1 M with dimethyl sulfoxide (DMSO) as stock solutions and stored in − 20 ℃ until use. The stock solutions were diluted to 10^–4^ M with 1 × Ringer’s buffer for ligand screening of PxylORs.

### Receptor expression in *Xenopus* oocytes and two electrode voltage clamp electrophysiological recording

*Xenopus* oocyte collection and preparation and electrophysiological recordings were conducted following previously reported protocols [60, 61]. The *PxylOR16*, *PxylOR27*, and *PxylOrco* cRNAs were synthesized using mMESSAGE mMACHINE T7 (Ambion, Austin, TX). *PxylOR16* or *PxylOR27* cRNAs (27.6 ng each) were simultaneously microinjected with *PxylOrco* cRNA into mature healthy *Xenopus* oocytes (stages V–VII). After injection, oocytes were incubated for 4–7 days at 16 ℃ in 1 × Ringer’s solution supplemented with 5% dialyzed horse serum, 50 μg/mL tetracycline, 100 μg/mL streptomycin, and 550 μg/mL sodium pyruvate. Odorant-induced currents were recorded with an OC-725C oocyte clamp (Warner Instruments, Hamden, CT) at a holding potential of − 80 mV. Oocytes were exposed to compounds in ascending order of concentration with an interval between exposures that allowed the current to return to baseline. The data were acquired and analyzed with Digidata 1440A and pCLAMP 10.2 software (Axon Instruments Inc., Union City, CA). Statistical comparison of responses of oocytes to tested ligands and dose–response data were analyzed using GraphPad Prism 5 (GraphPad Software Inc., San Diego, CA).

### Homozygous mutant generation by CRISPR/Cas9

To generate PxylOR16-knockout mutants, we selected a small sequence (GGCGCCCGAGCTGCAAGCTATCAAGG) as the single-guide RNA (sgRNA) target site in the second exon of PxylOR16. We have analyzed the possible off-target effects of this sgRNA in the *P. xylostella* genome and found that the sgRNA targets a single site, indicating that this guide has high specificity. sgRNA was synthesized according to the manufacturer’s procedures (GeneArt™Precision gRNA Synthesis Kit, ThermoFisher Scientific, Pittsburgh, PA, USA). For sgRNA synthesis, the upstream primer (TAATACGACTCACTATAG + target sequence) and the downstream primer (TTCTAGCTCTAAAAC + target sequence reverse complement) were spliced by PCR with a mixture of the Tracr fragment and the T7 primer to obtain the DNA template of the sgRNA. Then the sgRNA was synthesized by in vitro transcription. The PCR assembly reaction and in vitro transcription were conducted according to the manufacturer’s instructions. Finally, the DNA template was digested with DNase I and the sgRNA was cleaned using the sgRNA Clean Kit (ThermoFisher Scientific, Pittsburgh, PA, USA). The primer sequences are listed in Additional File 1: Table S2.

Based on the PxylOR16 genomic sequence, upstream (PxylOR16F) and downstream (PxylOR16R) primers were designed to detect mutants using PCR with genomic DNA as a template (Additional File 1: Table S2). Genomic DNA was extracted from adults using a TIANamp Genomic DNA Kit (Tiangen, China). PCR amplification products were purified on a 1.5% agarose gel, ligated into the pEASY-Blunt vector (TransGenBiotech, China), and sequenced by BGI (Beijing, China).

Freshly laid eggs (within 30 min) were used for microinjection. About 1 nL of a mixture of Cas9 nuclease (600 ng/µL, GeneArt Platinum Cas9 Nuclease, ThermoFisher Scientific, Pittsburgh, PA, USA) and sgRNA (600 ng/µL) was microinjected into each egg using a FemtoJet and InjectMan NI 2 microinjection system (Eppendorf, Hamburg, Germany). Approximately 500 eggs were microinjected, and 80 larvae hatched (G0). The adults of the G0 generation were hybridized to generate G1 individuals. When these G1 individuals grew into adults, we randomly grouped 50 pairs of parents and each pair was put in a plastic cup for mating and egg laying. As we should keep the insects alive for oviposition, genomic DNA was extracted from the whole bodies of the parents after oviposition for further genotyping by PCR and sequencing. One pair of G1 parents was selected because both the males and females exhibited heterozygous frame-shifting mutations (Fig. 2A), and their offspring was kept as G2. When the G2 individuals grew into adults, 10 pairs were randomly selected to perform single-pair mating and then genotyping by PCR and sequencing. The offspring of one pair of G2 parents of which both male and female carried the same heterozygous mutations were selected and kept as G3. When these G3 individuals grew into adults, 15 pairs were randomly selected to perform single-pair mating and then genotyping by PCR and sequencing. The offspring of one pair of G3 parents of which both male and female carried the same homozygous mutation were selected and kept as G4. The offspring of the G4 homozygous mutants were used for electrophysiological and behavioral analyses (Additional File 1: Fig. S4).

### EAG recordings

The responses of the antennae of virgin female and male moths (1–2 days after eclosion) to heptanal and trans-2-hexen-1-ol were analyzed by EAG recording. Heptanal and trans-2-hexen-1-ol were diluted in hexane at 1, 10, 100, and 1000 ng/μL. Test solutions (10 μL each) were deposited on a filter paper strip (1 × 5 cm) that was inserted into a Pasteur pipette. Hexane was used as a control. Each experiment was repeated 10 times. EAG responses for each compound were calculated by subtracting the EAG response to hexane. Data are reported as mean ± standard error of the mean (SEM), and means were compared by two-way ANOVA with Tukey’s pairwise test.

### Behavioral response trial

The behavioral responses of unmated *P. xylostella* adults to heptanal, body washes of parasitoid wasps, and live parasitoid wasps were tested using a Y-tube assay and those of larvae were tested using a Petri dish assay and a screening device. Heptanal was dissolved in paraffin oil at different concentrations (1, 10, 100, and 1000 ng/μL), and all bioassays were conducted in a temperature-controlled room at 26 ± 2 ℃. The adult and larvae preference indexes (PI) were calculated as described in previous studies in mosquitoes and *Drosophila* [62, 63] using the following equation: (*T* − *C*)/(*T* + *C*), where *T* is the number of choices for the treatment site and *C* is the number of choices for the control site.

The Y-tube consisted of a 15-cm stem and two 24.5-cm arms at a 45° angle with an interior diameter of 2.0 cm. The rate of airflow was 0.5 L/min and was controlled by connecting the olfactory arms to a vacuum pump. Before entering the tube, humidified continuous air was filtered through activated charcoal. The Y-tube was installed inside a behavior box (100 × 60 × 80 cm) equipped with a fluorescent lamp to provide light. A filter paper strip containing 10 μL of test solution was placed in the “treatment” arm, and a filter paper strip containing 10 μL of solvent was placed in the other arm as a control. Six moths were put one by one into the stem of the Y-tube for 10 min. During this time, moths entering one of the two arms more than 5 cm and staying there for more than 30 s were considered to have made a choice. Moths that did not make a choice were also recorded but the number was not considered in the analysis. Experiments were repeated 16 times for females and males separately. In Y-tube experiments with *B. pekinensis* leaves (1 × 1 cm) or intact *B. parachinensis* plants, leaves and plants were added in both arms and spiked with heptanal or paraffin oil as described above. The preference indexes of female and male moths were compared using Welch’s *t*-test analysis.

We used a simple Petri dish assay and a screening device to test the effect of heptanal, parasitoid wasp body washes, and live parasitoid wasps on *P. xylostella* third instar larvae. In the Petri dish assay (10 cm diameter × 1.5 cm height), two 1 × 1 cm pieces of fresh *B. pekinensis* leaves were placed at two opposite points of the Petri dish; one piece was spiked with 10 μL of test solution and the other with 10 μL of solvent alone as a control. After 4 h of starvation, 10 third instar larvae (all males or all females) were placed in one side of the Petri dish. The number of larvae on each piece of fresh *B. pekinensis* leaves at the opposite sides of the Petri dish was counted after 30 min [64]. The screening device (21 × 21 × 4 cm) consisted of three parts: traps A and B, a selection hole, and odor selection area. Traps A and B were each connected with *B. parachinensis* plants or live parasitoid wasps in glass jars, and traps captured the test larvae. The selection hole linked the trap and odor selection area, and there was a height difference between the trap and odor selection area, which prevented larvae from returning to the odor selection area. The odor selection area had a smaller height, which was conductive to the diffusion of odorant molecules, and a larger area, which provided sufficient activity space and improved the selection accuracy. The rate of airflow was 0.3 L/min and was controlled by connecting the screening device to a vacuum pump. Before entering the glass jars, humidified continuous air was filtered through activated charcoal. The release of test solution was performed in the same way as described for the adult Y-tube experiment (a filter paper strip containing 10 μL of test solution placed in the “treatment” jar and a filter paper strip containing 10 μL of solvent placed in the other jar as a control), with the presence a piece of *B. pekinensis* or an intact entire plant. Ten starved third instar larvae (all males or all females) were placed in the odor selection area of the screening device. The number of larvae in different sides of the screening device was counted after 2 h. Experiments were repeated 28 times (Petri dish assay) and 10 times (screening device) for each sex, and preference indexes were assessed using Welch’s *t*-test analysis.

We also compared the responses of the adults and larvae to larval-infected pants (*B. parachinensis*) and intact plants. Fifteen third instar *P. xylostella* larvae were placed on leaves of a *B. parachinensis* plant. Plants with no *P. xylostella* larvae (healthy plants) were used as a control. After 12 h of infestation, larvae and feces were removed and plants were placed in sealed glass jars. The behavioral responses of adults and larvae were tested using the Y-tube and the screening device, respectively, as described above. Experiments were repeated 16 times (adults) and 10 times (larvae) for each sex. The adult and larval preference indexes were calculated and analyzed by Welch’s *t*-test.

### Collection of plant volatiles and parasitoid cuticle volatiles

Fifteen third instar *P. xylostella* larvae were placed on leaves of a *B. parachinensis* plant. Plants with no *P. xylostella* larvae (healthy plants) were used as a control. After 12 h of infestation, larvae and feces were removed and plants were placed in sealed glass bottles. Volatiles from healthy and *P. xylostella* larvae-damaged plants were collected using solid-phase microextraction fibers (50/30-μm divinylbenzene/carboxen/polydimethylsioxane) for 4 h. Twenty wasps (male and female mixed) were extracted with dichloromethane (100 μL) for 30 min. The supernatants were removed, placed in a glass tube and stored at − 80 ℃ until use. The amount of heptanal in the wasp sample was evaluated by comparing the peak area to that of an internal standard (tetradecane, 100 ng diluted in 10 μL dichloromethane). Three replicates were performed for each collection types.

### Gas chromatography–mass spectrometry

All samples were analyzed by GC–MS using a LECO 4D GC × GC/TOF/MS equipped with a DB5/MS column (30 m × 0.25 mm ID × 0.25 μm film thickness; Agilent Technologies). The injector temperature was 270 ℃. The oven starting temperature of 40 ℃ was held for 9 min, followed by an increase of 10 ℃/min until a temperature of 270 ℃ was reached and then held for 2 min. The transfer line temperature was set at 250 ℃; the ion source temperature was 250 ℃. Ionization was by electron impact (70 eV), and the scan range was between m/z 50 and 350. Major volatile compounds were identified by comparison with the NIST 17 MS library. The presence of heptanal was confirmed by comparing the retention time and mass spectrum with those of synthetic heptanal.

### Statistical analysis

All data were calculated as mean ± SEM. Data statistics of PxylOR response spectra, dose responses, EAG, and adult and larvae behavioral experiments were assayed with SPSS Statistics 25.0 (IBM Corp, New York, NY, USA). GraphPad PRISM5.0 software (GraphPad Software, La Jolla, CA, USA) was used to draw graphics. The adult and larvae preference indexes were calculated as follows: (*T* − *C*)/(*T* + *C*), where *T* is the number of choices for the treatment site and *C* is the number of choices for the control site. Two-sample analysis was performed using Welch’s *t*-test (*a* = 0.05). Two-way analysis of variance (ANOVA) followed by least significant difference test were used to compare the EAG responses of WT and *PxylOR16*^*−/−*^ adults to heptanal or trans-2-hexen-1-ol.

### In situ hybridization

Digoxigenin-labeled sense and antisense probes of *PxylOR16* was generated from linearized, recombinant pSPT18 plasmids using the T7/SP6 RNA transcription system according to manufacturer’s protocol (Roche, Switzerland). Male and female antennae of 1- to 3-day-old moths were embedded in Jung tissue-freezing medium and frozen at − 60 °C rapidly. Longitudinal Sects. (10 μm) of antennae were thaw-mounted on anti-off slides using a Cryostar NX50 cryostat (ThermoFisher Scientific, Pittsburgh, PA, USA) and air-dried for 30 min at room temperature. In situ hybridization was performed as described in previous reports [65, 66].

### Supplementary Information

**Additional file 1: Fig. S1** Tissue expression patterns of all putative *Plutella xylostella* OR genes. The cDNA templates for PCR analyses were from larval heads (1st, 2nd, 3rd female, 3rd male, 4th female and 4th male instar larvae) and adult antennae (male adults: MA, female adults: FA). W: water control. Seven PRs (*PxylOR1*, *PxylOR3*, *PxylOR4*, *PxylOR5*, *PxylOR6*, *PxylOR7*, and *PxylOR41*) were specifically or highly expressed in the male antennae. **Fig. S2** Attraction of wild-type and PxylOR16 mutant of *Plutella xylostella* to sex pheromone (Z-11-hexadecenal). **Fig. S3** Location of *PxylOR16* in male and female adult antennae of* Plutella. xylostella*. **Fig. S4** Diagram of the crossing strategy used to obtain the homozygous knockout strain. The genotype (5-nt insertion and 1-nt deletion) was marked in red, while other mutant genotypes were marked in blue. **Table S1.** Plant volatiles used in this study. **Table S2.** Primers used in this study.

## Data Availability

All data generated or analyzed during this study are included in this published article and its supplementary information files.

## Abbreviations

ANOVA: Two-way analysis of variance
EAG: Electroantennography
GC-EAD: Gas chromatography–electroantennographic detection
GC/MS: Gas chromatography/mass spectrometry
IPM: Integrated pest management
OBP: Odorant binding protein
OR: Odorant receptor
Orco: Odorant receptor co-receptor
PR: Pheromone receptor
