# Supplementary material for: An odorant receptor mediates the avoidance of Plutella xylostella against parasitoid
Source: BMC Biol. 2024 Mar 13;22:61. doi: 10.1186/s12915-024-01862-9 (PMC10935965; doi:10.1186/s12915-024-01862-9)
Supplement: Supplementary file 1 — Additional file 1: Fig. S1 Tissue expression patterns of all putative Plutella xylostella OR genes. The cDNA templates for PCR analyses were from larval heads (1st, 2nd, 3rd female, 3rd male, 4th female and 4th male instar larvae) and adult antennae (male adults: MA, female adults: FA). W: water control. Seven PRs (PxylOR1, PxylOR3, PxylOR4, PxylOR5, PxylOR6, PxylOR7, and PxylOR41) were specifically or highly expressed in the male antennae. Fig. S2 Attraction of wild-type and PxylOR16 mutant of Plutella xylostella to sex pheromone (Z-11-hexadecenal). Fig. S3 Location of PxylOR16 in male and female adult antennae of Plutella. xylostella. Fig. S4 Diagram of the crossing strategy used to obtain the homozygous knockout strain. The genotype (5-nt insertion and 1-nt deletion) was marked in red, while other mutant genotypes were marked in blue. Table S1. Plant volatiles used in this study. Table S2. Primers used in this study. [file 12915_2024_1862_MOESM1_ESM.docx]

**Supplementary Material**

**An Odorant Receptor Mediates the Avoidance of *Plutella xylostella* Against Parasitoid**

Yipeng Liu^1,2,3^, Sai Zhang^1^, Song Cao^1^, Emmanuelle Jacquin-Joly^4^, Qiong Zhou^5^, Yang Liu^1,*^, Guirong Wang^1,2,*^

^1^ State Key Laboratory for Biology of Plant Diseases and Insect Pests, Institute of Plant Protection, Chinese Academy of Agricultural Sciences, Beijing, 100193, China;

^2^ Shenzhen Branch, Guangdong Laboratory of Lingnan Modern Agriculture, Genome Analysis Laboratory of the Ministry of Agriculture and Rural Affairs, Agricultural Genomics Institute at Shenzhen, Chinese Academy of Agricultural Sciences, Shenzhen, 518120, China;

^3^ Zhejiang Provincial Key Laboratory of Biometrology and Inspection and Quarantine, College of Life Sciences, China Jiliang University, Hangzhou, 310018, China;

^4^ Institute of Ecology and Environmental Sciences of Paris, Sorbonne Université, INRAE, CNRS, IRD, UPEC, UniversitéParis Cité, Versailles, 78026, France;

^5^ College of Life Sciences, Hunan Normal University, Changsha, 410006, China;

**^*^** Correspondence author: Yang Liu, [yangliu@ippcaas.cn](mailto:yangliu@ippcaas.cn); Guirong Wang, [wangguirong@caas.cn](mailto:wangguirong@caas.cn)

**Additional file 1:**

**Fig. S1** Tissue expression patterns of all putative *Plutella xylostella* OR genes.

**Fig. S2** Attraction of wild-type and PxylOR16 mutant of *Plutella xylostella* to sex pheromone (Z-11-hexadecenal).**Fig. S3** Location of *PxylOR16* in male and female adult antennae of *Plutella xylostella*

**Fig. S4** Diagram of the crossing strategy used to obtain the homozygous knockout strain.

**Additional file 2:**

**Table S1.** Plant volatiles used in this study.

**Table S2.** Primers used in this study.

**
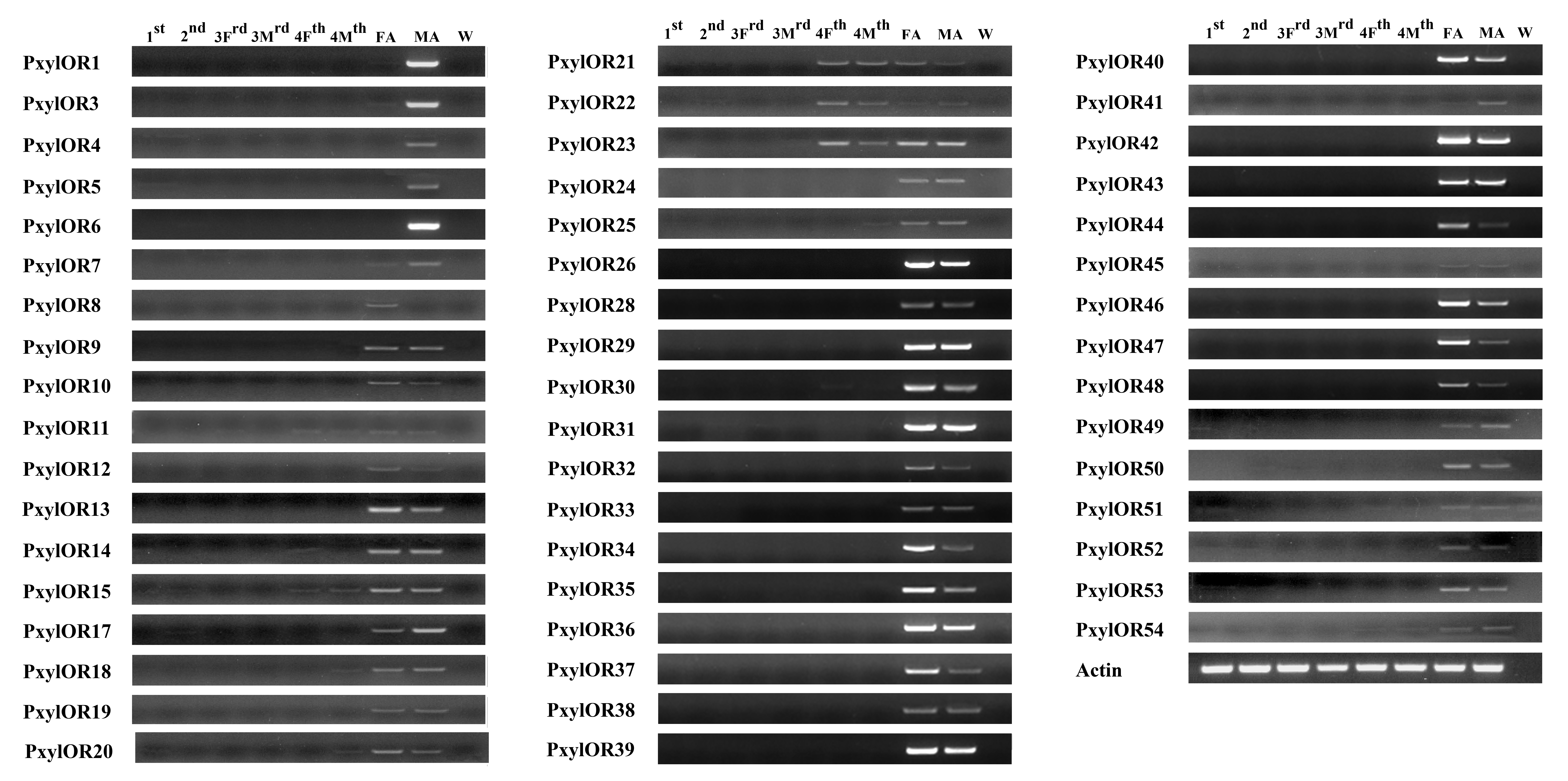
**

**Fig. S1** Tissue expression patterns of all putative *Plutella xylostella* OR genes. The cDNA templates for PCR analyses were from larval heads (1^st^, 2^nd^, 3^rd^ female, 3^rd^ male, 4^th^ female and 4^th^ male instar larvae) and adult antennae (male adults: MA, female adults: FA). W: water control. Seven PRs (*PxylOR1*, *PxylOR3*, *PxylOR4*, *PxylOR5*, *PxylOR6*, *PxylOR7*, and *PxylOR41*) were specifically or highly expressed in the male antennae.


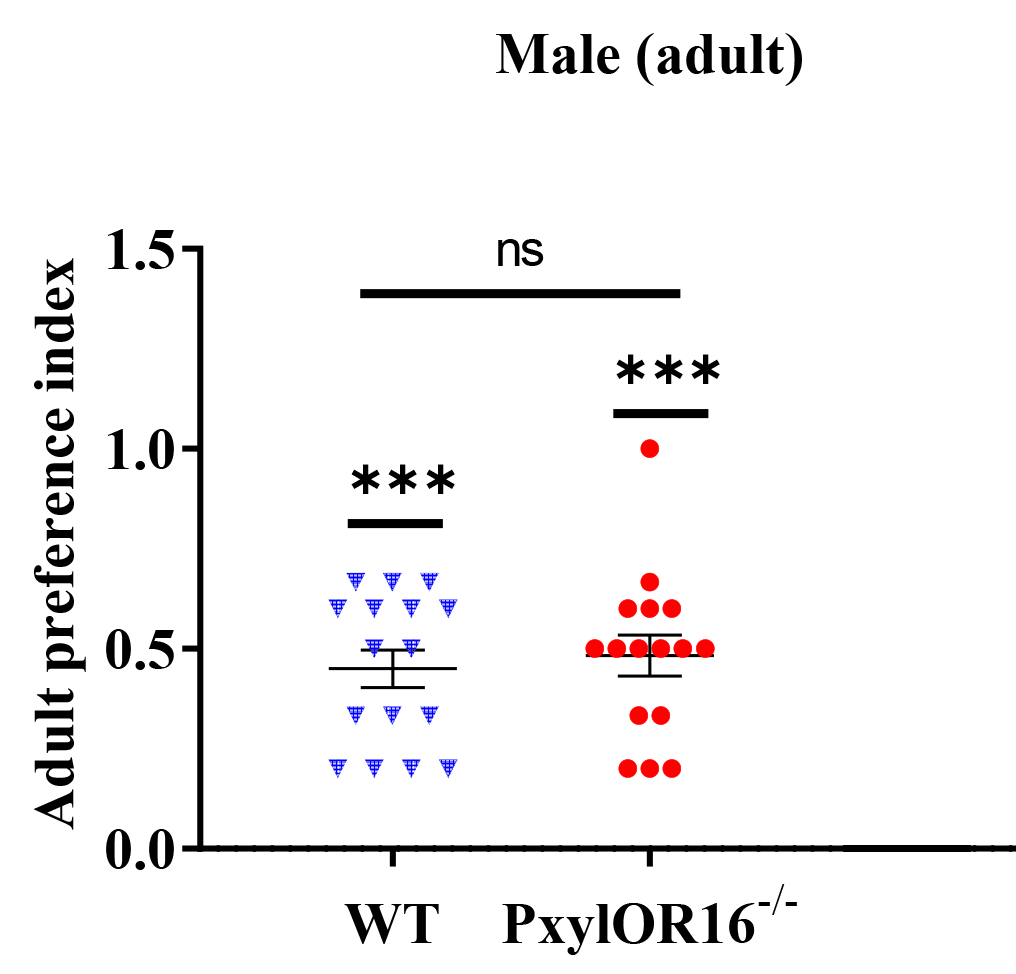


Fig. S2 Attraction of wild-type and PxylOR16 mutant of *Plutella xylostella* to sex pheromone (Z-11-hexadecenal).


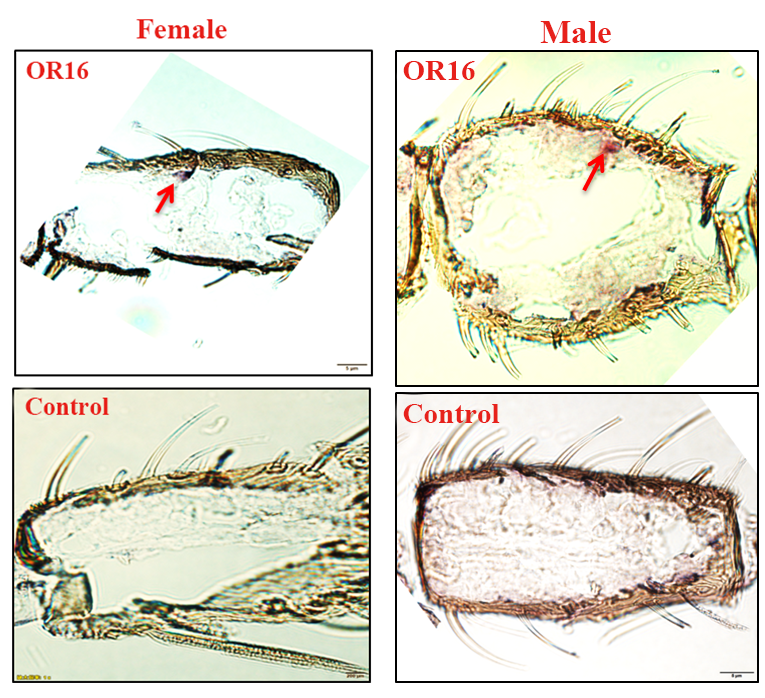


Fig. S3 Location of *PxylOR16* in male and female adult antennae of *Plutella xylostella*.

**
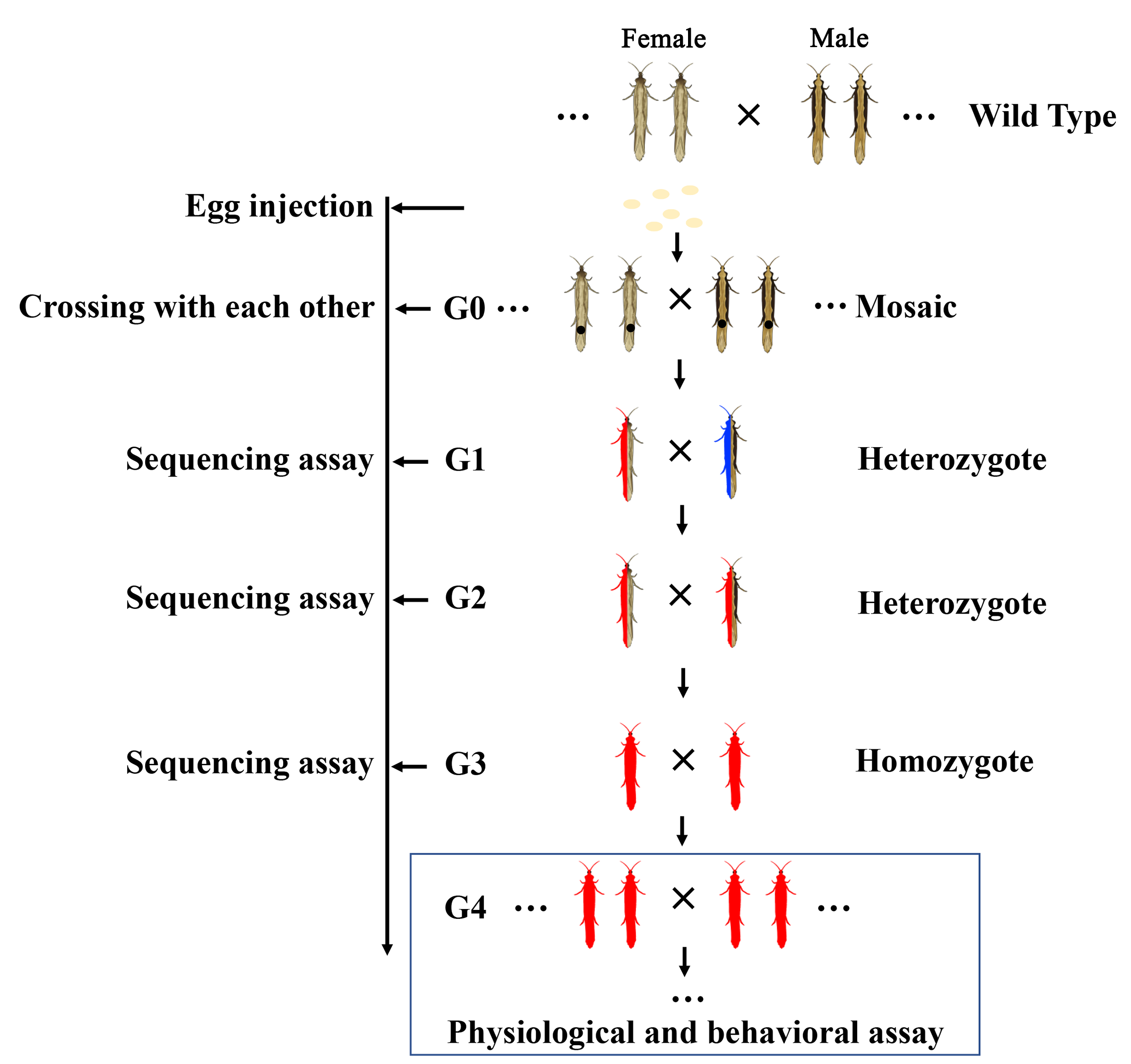
**

**Fig. S4** Diagram of the crossing strategy used to obtain the homozygous knockout strain.

**Table S1. Plant volatiles used in this study**

| **No.** | **Name** | **CAS no.** | **No.** | **Name** | **CAS no.** |
| --- | --- | --- | --- | --- | --- |
| 1  2  3  4  5  6  7  8  9  10  11  12  13  14  15  16  17  18  19  20  21  22  23  24  25  26  27  28  29  30  31  32  33  34  35  36 | 2-Phenylethanol  cis-3-Hexen-1-ol  β-Citronellol  Geraniol  cis-2-Hexen-1-ol  1-Heptanol  (1S)-(-)-Verbenone  1-Hexanol  trans-3-Hexen-1-ol  3, 7-Dimethyl-3-octanol  (-)-Borneol  (+)-Borneol  (1R)-(-)-Myrtenol  (-)-trans-Pinocarveol  (-)-Linalool  Linalool  Methyl benzoate  Myrcene  α-Pinene  (-)-β-Pinene  Camphene  α-Humulene  α-Terpinene  (-)-trans-Caryophyllene  (-)-Caryophyllene oxide  Farnesene  trans-2-Hexen-1-al  4-Ethylbenzaldehyde  3-Vinylbenzaldehyde  (1R)-(-)-Myrtenal  Benzaldehyde  Heptanal  trans-2-Hexenyl acetate  cis-3-Hexenyl acetate  1, 4-Diethylbenzene  4′-Ethylacetophenone | 60-12-8  928-96-1  106-22-9  106-24-1  928-94-9  111-70-6  1196-01-6  111-27-3  928-97-2  78-69-3  464-45-9  464-43-7  19894-97-4  547-61-5  126-91-0  78-70-6  93-58-3  123-35-3  80-56-8  18172-67-3  79-92-5  6753-98-6  99-86-5  87-44-5  1139-30-6  502-61-4  6728-26-3  4748-78-1  19955-99-8  18486-69-6  100-52-7  111-71-7  2497-18-9  3681-71-8  105-05-5  937-30-4 | 37  38  39  40  41  42  43  44  45  46  47  48  49  50  51  52  53  54  55  56  57  58  59  60  61  62  63  64  65  66  67  68  69  70  71 | Allyl isothiocyanate  Benzyl acetate  Tetradecane  Nonyl acetate  Hexyl acetate  Ocimene  Tridecane  Methyl Salicylate  (±)-Camphor  Nerolidol  2, 6-Di-tert-butylphenol  1-Aminoanthracene  2-Pentadecanone  Acetophenone  Cumene  (+)-Cedrol  Octyl acetate  Benzaldehyde  Ethyl butyrate  Ethyl hexanoate  β-Ionone  (S)-cis-Verbenol  Trans-2-Hexen -1-ol  Decane  trans-2-Deceneol  1-Cetanol  2-Octyldecanol  Hexadecane  (S)-(-)-Limonene  Hexacosane  2-Hexadecanol  Mesitylene  Heptylether  Tritetracontane  Heptadecane | 57-06-7  140-11-4  629-59-4  143-13-5  142-92-7  13877-91-3  629-50-5  119-36-8  76-22-2  7212-44-4  128-39-2  610-49-1  2345-28-0  98-86-2  98-82-8  77-53-2  112-14-1  100-52-7  105-54-4  123-66-0  14901-07-6  18881-04-4  928-95-0  124-18-5  18409-18-2  36653-82-4  45235-48-1  544-76-3  5989-54-8  630-01-3  14852-31-4  108-67-8  629-64-1  7098-21-7  629-78-7 |

**Table S2. Primers used in this study**

| **Primers for RT-PCR** | | |
| --- | --- | --- |
|  | Forward Primer | Reverse Primer |
| PxylOrco | CTCACCGCCAACACCATCAC | CAGCAGCCACGAGCAGAACAT |
| PxylOR1 | CTCCCACTTCTTCACCATGTACC | CATCCTCCCAACTATCACCATC |
| PxylOR3 | GCTGAGATTTCTGCGTATTGGG | ACGCAGATGCTACACGCAGTTAT |
| PxylOR4 | GCCCAGGACTTGCTCTTGTC | CAGGGTTTCGGGATCAGGTC |
| PxylOR5 | TGTTATCACAAGCACAAGGGAA | ATTCATCGTCGTAGATATGTAGAAGTG |
| PxylOR6 | ATGCAGATGACGCTGATGGTA | TCAATGGAGCAAACTGACACG |
| PxylOR7 | TTGTGGCGTCACTCACTGTTC | TTGTAACTGTTGAATATCGGTATTCC |
| PxylOR8 | GATCGGTCATGTTTACATTACGAC | TATTAGGAGCAAATCCAGAGTGC |
| PxylOR9 | GCTTCAGTATTAGTCGTGGCA | AATATCTGTCGGAGAAAGGAGA |
| PxylOR10 | GCACGCATTGCAGGAACAAGA | GGACACCACCGACAGCAGGAT |
| PxylOR11 | ATAGGGATATGATGCGTCACAAGG | GTGCGGTAGTCAAACGGGAGT |
| PxylOR12 | AAGAATCTGATCGCGTTCACC | CCAGGAATAGGCAGCAAAGTA |
| PxylOR13 | GCCGTGTTGGTTTGGAACTC | ACTGCATGTGACGCAGAAGA |
| PxylOR14 | TTTTAGTGTTTCTATACGTGCTGG | AGTCCTCACTGAGCGTGTTGT |
| PxylOR15 | GGCGTCTCAACCCATATCCAC | CACCAGCAAATACATCAGTGTCCA |
| PxylOR16 | CCCGACCACTACCGAGATA | GCTGAGGACGATATTCACAAGA |
| PxylOR17 | GCACTTATTTATCCCAACCCG | GACCAACCCTTTCTTTATGTATG |
| PxylOR18 | AATTACCGGCATTTGGTTTCG | TTCACCGATAAATATGTCACTGTCC |
| PxylOR19 | CCAAAGCGGATATTGAAGCC | GCGTTGGTGTCATAGGAGGAG |
| PxylOR20 | TCTACTTTCTTTACGATGGGCTTCT | ACCCAATACGAAGTACGCACG |
| PxylOR21 | TACATTCTCGTTACTAATCGCAGAC | GGTAAACATTCCACCTTCCCT |
| PxylOR22 | TCTGTGGCCGCTTATTTTACA | CAGGCCCATGCTACAGTGTTA |
| PxylOR23 | CGGTTGGAAAGTGTTAGCCCTGTG | GTTTCGCCCGCAAATTCATCG |
| PxylOR24 | CTGTCGTCTTGCGGTACATCC | GGGTAAACAATTAACTTCCCTTTC |
| PxylOR25 | CTGATCCCGAGTTTCCATCCT | AAATGCCAGTTTCGTCAATCC |
| PxylOR26 | CTTCTGGCTCCTAACCTGTGG | CGAAATTGTCGCTCAACTCTG |
| PxylOR27 | TATCGGCACTTACAACATCCG | CATTAGCAGGCTCGTCATTCG |
| PxylOR28 | GAAGCTCCTAAATACTATGAAAGG | TTAATCTCAACGGGAAACAAC |
| PxylOR29 | CAACCTCATTGGTCCAGACAC | CTCCGAACAGGCAAATAAGAA |
| PxylOR30 | GCTTATCGCAAGCTACGCG | GTGACCGTGAAAAATGAGTATAG |
| PxylOR31 | ACCACGCAGATACGGATACTGA | AGCTCCACGACTATGCACGAC |
| PxylOR32 | TTGCCTCTACTGCTGATGGTG | AATGAACCCGCTCAGACAAAT |
| PxylOR33 | GTCCAGTATCGTCCTCAACGGC | TGAAAGCGGTCATGCAAACAA |
| PxylOR34 | TCTCCCTATGGCTCAATGTAAC | CACCGAGGACTCACTCAACTC |
| PxylOR35 | CAATTATTATTTCTTGAGCGACGAG | CAGTAGCAAGGAGGCGAGGAT |
| PxylOR36 | CTTTGAGGATTCTGGCGTTTG | AGATATGGCGATCTTGGTGGA |
| PxylOR37 | GGCAATAATCTGGTACAAGCAGG | CCGCTACAAACTTCAAACGCATAA |
| PxylOR38 | TATACGTGAATGGGTGGGACG | CCTCCAAACAGTGTTGCTGCTA |
| PxylOR39 | TAGACATCGAGGCCCTTTACC | GCACTTCAGTTTCAGGAGCAA |
| PxylOR40 | ACTCTGCCATTTCATTCATGTTTCT | AGCTCGTAAGCTGGTGACTTCG |
| PxylOR41 | CCCACTATTTCACTGCTACGACC | GCAAACCTTACTATTTCCCGATG |
| PxylOR42 | CATACGAGTTTTCGGTGATTTG | ATGACCATGCAAAGAGCTACAC |
| PxylOR43 | AGAAACGTCTTTGGACCACCT | CACTGCTCTTATCACCTGCTCAC |
| PxylOR44 | TCGGAGTGCCCATCATGTTCT | CCCGTCGTAAGCCGTGTAGC |
| PxylOR45 | GTCAAATTGAAGCCATGTTGATC | CGGCGACTGTGGTACTGTAGG |
| PxylOR46 | AGGATACAGCCAGATGAAACG | TTGCTCACATACAGCACGATG |
| PxylOR47 | GCAGGTGATCCTGTGGCTCTA | TGCTTTCTCCTTGCTCGTCTC |
| PxylOR48 | AACGATGGAAGGGAGTGGAGA | AGGCGTGTTGATGAGATGTAGC |
| PxylOR49 | TGTGCGGATGCTCCAAGTGAG | GTGGTTGGCGAGGATTTCCCT |
| PxylOR50 | TACGTGCCGTTCGATAAATACAGT | GGCGAGCTGGCTCTTACAAAG |
| PxylOR51 | CGGCTACTGGCTCTACGTGCTG | CCCGCCTTAGTGAGTTGAATGG |
| PxylOR52 | CGCCTAATACAGAGTCTGGACGA | GTAGAATGCTCAGTTGAGTTTTGCT |
| PxylOR53 | TTCGTTGGCATTTCCATCTTT | CAACAGCACCCTCACAAACCT |
| PxylOR54 | GCGAAATCTTCAATTATTTGCC | TGGTTGTGTCTGGTCCCTGG |
| PxylActin | GCCGTCTTCCCGTCCAT | GATACCTCTCTTGCTCTGGGC |
| **Specific primers for cloning** | | |
| PxylOR16F | G*ACTAGT*GCCACCATGTCGGTCGATCCGACGGT (*Spe* I) | |
| PxylOR16R | ATTT*GCGGCCGC*TTAGTTTTCCTTATACATTGTG (*Not* I) | |
| PxylOR27F | G*ACTAGT*GCCACC ATGGAGCCGAGCAAGGTTC (*Spe* I) | |
| PxylOR27R | ATTT*GCGGCCGC* TTACATCATATCGTGAAGCATTGTA (*Not* I) | |
| **Specific primers for sgRNA** | | |
| PxylOR16F | GAAATTAATACGACTCACTATAGCCCGAGCTGCAAGCTATCA | |
| PxylOR16R | TTCTAGCTCTAAAACTGATAGCTTGCAGCTCGGGC | |
| **Detection of the PxylOR16 cluster deletion** | | |
| PxylOR16F | GTAGCTTCAAGCTGTGGTCCGTGTGG | |
| PxylOR16R | CCGGAGAGGATCTCGAACAGGTACAC | |

The restriction enzyme site added to each primer is indicated in parenthesis after the sequence, and the cutting sites are in italics.
